# Validation of a questionnaire of knowledge and attitudes about the subcutaneous venous reservoir in nursing*

**DOI:** 10.1590/1518-8345.3255.3250

**Published:** 2020-04-17

**Authors:** Roberto Raña-Rocha, Ignacio López-de-Ullibarri, María-Jesús Movilla-Fernández, Carmen Coronado Carvajal

**Affiliations:** 1 University of A Coruña, Health Sciences Department, Research Group GRINCAR, Ferrol, Galicia, Spain.; 2 University of A Coruña, Mathematics Department, Research Group MODES, CITIC, Ferrol, Galícia, Spain.

**Keywords:** Vascular Access Devices, Surveys and Questionnaires, Reproducibility of Results, Health Knowledge, Attitudes, Practice, Nursing Care, Nursing Clinicians, Dispositivos de Acesso Vascular, Inquéritos e Questionários, Reprodutibilidade dos Testes, Conhecimentos, Atitudes e Prática em Saúde, Cuidados de Enfermagem, Enfermeiras Clínicas, Dispositivos de Acceso Vascular, Encuestas y Cuestionarios, Reproducibilidad de los Resultados, Conocimientos, Actitudes y Práctica en Salud, Atención de Enfermería, Enfermeras Clínicas

## Abstract

**Objective:**

design and validate a questionnaire to evaluate the knowledge and attitudes of nurses about the subcutaneous venous reservoir.

**Method:**

pilot test: 30 specialized care nurses. Main study: 236 nurses of primary and specialized care. Content validity was evaluated by Lawshe index, reliability by test-retest, internal consistency by Cronbach alpha, and construct validity by exploratory factorial analysis.

**Results:**

Items with a Lawshe index lower than 0.51 were eliminated. In the test-retest, the intraclass correlation coefficient was higher than 0.75 for all items. The Cronbach alpha of the attitude questionnaire reached 0.865. The Cronbach alpha value for knowledge was 0.750. The exploratory factor analysis identified a set of four dimensions for each part that explain 64% (attitude) to 80% (knowledge) of variability.

**Conclusion:**

the analysis of the reliability and validity of the questionnaire supports its use as an instrument to assess the knowledge and attitudes of nurses towards the subcutaneous venous reservoir.

## Abstract (pt)

**Objetivo:**

construir e validar um questionário para avaliar os conhecimentos e as atitudes dos profissionais de enfermagem sobre o cateter venoso totalmente implantado.

**Método:**

teste piloto: 30 enfermeiros de atendimento especializado. Estudo principal: 236 enfermeiros de atendimento primário e especializado. Avaliou-se a validade de conteúdo por meio do índice de Lawshe, a confiabilidade por meio do teste-reteste, a consistência interna mediante o alfa de Cronbach, e a validade de construto por meio de uma análise fatorial exploratória.

**Resultados:**

foram eliminados os itens com um índice de Lawshe inferior a 0,51. No teste-reteste, o coeficiente de correlação intraclasse foi maior que 0,75 para todos os itens. O alfa de Cronbach do questionário de atitude atingiu um valor de 0,865. O valor de alfa de Cronbach para o de conhecimentos foi de 0,750. A análise fatorial exploratória identificou um conjunto de quatro dimensões para cada parte, que explicam a variabilidade de 64% (atitude) a 80% (conhecimentos).

**Conclusão:**

a análise da confiabilidade e a validade do questionário endossam seu uso como instrumento para avaliar os conhecimentos e as atitudes dos profissionais de enfermagem em relação ao cateter venoso totalmente implantado.

## Abstract (es)

**Objetivo:**

diseñar y validar un cuestionario para evaluar los conocimientos y las actitudes del personal de enfermería sobre el reservorio venoso subcutáneo.

**Método:**

prueba piloto con 30 enfermeros/as de atención especializada. Estudio principal con 236 enfermeros/as de atenci*ón primaria y especializada*. Se evaluó la validez de contenido mediante el índice de Lawshe, la fiabilidad mediante test-retest, la consistencia interna mediante el alfa de Cronbach y la validez de constructo con un análisis factorial exploratorio.

**Resultados:**

se eliminaron los ítems con un índice de Lawshe inferior a 0,51. En el test-retest, el coeficiente de correlación intraclase resultó ser mayor que 0,75 para todos los ítems. El alfa de Cronbach del cuestionario de actitud alcanzó un valor de 0,865. El valor de alfa de Cronbach para el de conocimientos fue de 0,750. El análisis factorial exploratorio identificó un conjunto de cuatro dimensiones para cada parte que explican del 64% (actitud) al 80% (conocimientos) de la variabilidad.

**Conclusión:**

el análisis de la fiabilidad y la validez del cuestionario avalan su uso como instrumento para valorar los conocimientos y actitudes del personal de enfermería en torno al reservorio venoso subcutáneo.

## Introduction

The subcutaneous venous reservoir (SVR) is a type of central venous access fully implanted under the skin, consisting of a catheter and a component fixed by suture, suitable for children and adults, which ends in the superior vena cava or the right atrium and allows the administration of various therapeutic measures improving the quality of life of patients^(^ 1 ^-^ 3 ^)^. The application of prolonged therapeutic guidelines intravenously for the treatment of various pathologies leads to a decrease in the patient’s vascular network^(^ 4 ^)^. That is why SVR is used and this fact implies that more and more nursing professionals are daily confronted with these devices, both in primary care and specialized care, and therefore competence in the management of SVR is necessary^(^ 5 ^)^.

The SVR should be considered the first choice for two reasons: the comfort it gives to the patients by avoiding the traumatic search for a vein, decreasing their level of anxiety and increasing their comfort, which improves their quality of life; and the care work of the professional who handles it by allowing a fast and safe venous access^(^ 6 ^-^ 7 ^)^. A misuse of SVR can cause irreparable damage in it and therefore lead to the need for a replacement of the central access and damage to both the costs and the patient’s quality of life^(^ 8 ^-^ 10 ^)^. It is essential to nursing staff handling these devices safely, requiring specific knowledge and attitudes^(^ 11 ^)^.

Knowledge regarding the attitudes and level of knowledge of nursing professionals concerning the management of SVR is limited, and there are clear evidences about the problems in the use of these devices by the professional Nursing group. This situation also affects the comfort of patients with SVR^(^ 12 ^-^ 19 ^)^. The available studies mainly refer to the technique of implantation of the different devices and catheters, to the technique of handling the SVR, and to the complications associated with it. We have not found any validated studies or tools that would allow us to obtain specific results about the level of knowledge and attitudes of the nursing professional towards the use of SVR. We believe it is necessary to develop a validated tool to measure the level of knowledge and attitudes of the nursing professionals towards the use of SVR, both in primary care and in specialized care. The use of this new tool will allow us to conduct studies about the nursing professionals in our environment in terms of knowledge and attitudes regarding the use of SVR. Our findings may have an impact on the improvement of the quality of care received by patients who use this device.

The objective of this study was to design and validate a questionnaire to evaluate the knowledge and attitudes of nurses about the subcutaneous venous reservoir.

## Method

A cross-sectional descriptive study was conducted between the months of November 2016 and October 2017.

A bibliographic search in the PubMed database was performed using the following combination of descriptors:

(((((“Nurses”[Mesh]) OR “Nurse Clinicians”[Mesh] OR “nurs*” [tw])) AND (“Vascular Access Devices”[Mesh] OR “subcutaneous reservoir” [tw])) AND (“Surveys and Questionnaires”[Mesh] OR “survey*” [tw] OR “questionnaire*” [tw]))

This search was adapted and executed in the databases Scopus, Web of Sciences, Cinahl and Dialnet.

**Figure 1:**
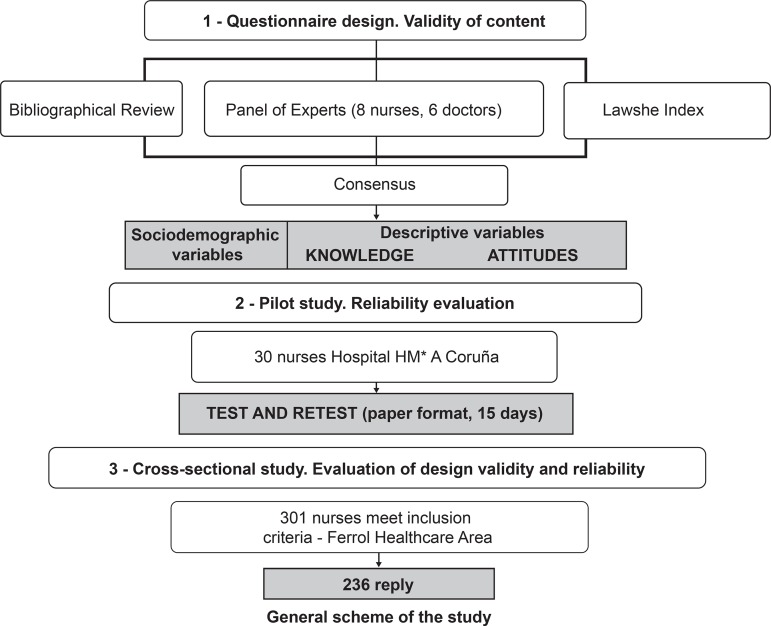
General scheme of the study. A Coruña and Ferrol, Spain, 2017 *HM = Madrid Hospitals

The development of the study consisted of three phases^(^ 20 ^-^ 21 ^)^ (Figure 1).

The participants were selected among the health professionals of the Hospital HM (*Hospitales Madrid* - Madrid Hospitals) in A Coruña and the Health Area of Ferrol (*Servicio Gallego de Salud* - Gallician Health Service).

For the pilot test all the nursing staff of the Hospital HM Modelo in A Coruña were included. For the cross-sectional study, the nursing staff of the Ferrol Health Area of primary care of all the health centers and specialized care of medical-surgical units were included. Rehabilitation units, psychiatry, external consultations and central services were excluded (because the SVR is not used in these units), operating room (due to the difficulty in access) and oncology day hospital (because there is a collaborating researcher in this unit).

The bibliographic search described was used to define the content of the instrument. A questionnaire structured in two blocks was developed: knowledge items and attitude items. The knowledge block initially consisted of 15 politomic answer questions (yes/no/not-know-not answered) that focused on management techniques and recommendations for the device. The attitude block was initially composed of 16 questions on a 5-point Likert scale (from “totally disagree” to “totally agree”).

A panel of experts was organized to validate the content of the questionnaire with participation of 8 nurses (Oncology, Hematology and Day Hospital) and 6 doctors (Hematology, Oncology and Vascular Surgery) from the Hospital *HM Modelo*, with extensive knowledge about the theoretical framework of SVR and experience in its management.

To validate the content of the questionnaire, a set of aspects was considered in the evaluation of each question. In the attitude questionnaire, the four aspects were “Apprehension of the professional managing the SVR”, “Fear of the professional to make mistakes managing SVR “, “Safety in the handling of SVR by the professional” and “Extrinsic hindering element for the use of SVR”. In the knowledge questionnaire “Knowledge about the applications of SVR”, “Knowledge about the handling of SVR”, “Training on SVR” and “Quality of care provided to the patient” were evaluated. In addition, in each question a section of observations was included so that the experts could contribute.

Once the 14 questionnaires were collected, Lawshe’s content validity index (CVI) was used to evaluate each item. In each of them, the four aspects proposed for that block of the questionnaire had to be evaluated individually as “not relevant”, “ little relevant”, “quite relevant”, “very relevant”. As a criterion for excluding items from the questionnaire, it was decided to eliminate those that did not obtain a score greater than 0.51 in at least one of the aspects^(^ 22 ^)^. The questionnaire was repeated by eliminating the items that did not pass the CVI filter and including the suggestions of the group of experts.

The questionnaire was conducted between April and May 2017 in the HM Modelo group, selecting a simple random sample of 30 nurses carried out by the hospital Management from the staff list. The questionnaire was completed by 100% of the professionals. The tool was used in paper format and was completed in an average time of approximately 15 minutes, without any problem during the process. It was administered on two occasions, spaced in 15 days^(^ 23 ^)^.

Reliability was evaluated by measuring internal consistency with the Cronbach alpha index. In addition, the test-retest method was used by calculating the intraclass correlation coefficient (ICC) as a measure of reliability^(^ 24 ^-^ 25 ^)^.

Due to the impossibility of randomizing the sample selection because of compliance with Organic Law 15/1999, of 13 December, of the Protection of Personal Data^(^ 26 ^)^, an attempt has been made to obtain a sample of the largest possible size. The questionnaire was administered by hand to a total population of 301 nursing professionals (in collaboration with supervising nurses and coordinators) of the Ferrol Health Area: primary care nurses (125); specialised care nurses (176).

In addition, to study the internal consistency again, an exploratory factorial analysis (EFA) was performed to assess construct validity, which was carried out from the polychoric correlation matrix. The factors were extracted by a generalized least squares method, applying an oblique rotation of the factors. The selection of the number of factors was made by means of the joint evaluation of the shape of the scree graph, the magnitude of the eigenvalues with respect to a threshold of 1 (considered loosely, to allow for sampling error), and the interpretability of the factors retained.

For statistical analysis, we used the program IBM^®^ (International Business Machines) SPSS (Statistical Package for the Social Sciences) Statistics^®^ and, for EFA, the psych package of R.

To carry out the study, the confidentiality of the information has been guaranteed according to the regulations in force^(^ 26 ^)^ and the ethical principles of the Declaration of Helsinki. The mention of the city and the institution does not enable identifying the participants in the study. The study received the approval of *Comité de Ética de Investigación de Galicia* (Research Ethics Committee of Galicia) from 05/20/2015 and protocol number 2014/173 and of *Comité Ético de Investigación Clínica del Grupo Hospitales Madrid* (GHM - Hospitals Madrid Group) from 12/18/2015 and protocol number 15.12.899-GHM, as well as permission from *Gerencia de Gestión Integrada de Ferrol*, *Servicio Gallego de Salud* (SERGAS - Integrated Management Agency of Ferrol, Galician Health Service) from 02/10/2015. The participants consented to their participation in the study protecting the questionnaire as they were informed in the head of the instrument.

## Results

Table 1 shows the minimum, maximum and median CVI values for each item of the attitude and knowledge questionnaires in the initial tool. Two questions were removed from the initial knowledge questionnaire because of the DVI calculation. The new version of the questionnaire was reduced to a knowledge block with 13 questions and an attitude block with 16.

**Table 1:** Contents validity index. A Coruña, Spain, 2017

Attitude Questionnaire				Knowledge Questionnaire		
Item	Minimum value	Maximum value	Median value	Minimum value	Maximum value	Median value
1	0.4285	0.5714	0.5356	0.4285	0.7142	0.6070
2	0.4285	0.7142	0.5356	0.4285	1	0.7856
3	0.1428	1	0.5356	0.1428	0.4285*	0.2856
4	0.4285	0.8571	0.6428	0.5714	0.8571	0.7499
5	0.2857	0.8571	0.5356	1	1	1
6	0.1428	0.7142	0.5356	0.5714	1	0.8214
7	0.2857	0.7142	0.5713	0.7142	1	0.9285
8	0.2857	0.8571	0.5356	0.7142	1	0.8571
9	0.1428	1	0.6428	0.2857	0.5714	0.3928
10	0.2857	0.8571	0.5356	0.4285	0.7142	0.6427
11	0.1428	0.8571	0.5713	0	0.4285*	0.2499
12	0	1	0.5356	0.1428	1	0.5357
13	-0.8571	0.7152	-0.3571	0.5714	0.8571	0.7499
14	-0.5714	0.5714	-0.1428	0.4285	0.7142	0.6427
15	0.2857	0.8571	0.5356	0.5714	0.8571	0.6785
16	0.1428	0.8571	0.5356			

* Maximum values obtained in the calculation of the Content Validity Index lower than the minimum required (0.51)

In the test-retest carried out for the reliability assessment in the pilot study, the ICC was greater than 0.75 for all items (Table 2). Cronbach’s alpha, calculated with data from the first administration of the test, was 0.818 for the attitude questionnaire and 0.608 for the knowledge questionnaire. Similar values were obtained in the retest (0.819 and 0.642, respectively).

**Table 2:** Intraclass Correlation Coefficient and 95% Confidence Intervals. A Coruña, Spain, 2017

	Attitude Questionnaire			Knowledge Questionnaire		
Item	ICC*	IC^†^		ICC*	CI^†^	
1	0.793	0.610	0.896	0.880	0.763	0.941
2	0.952	0.902	0.977	0.786	0.598	0.892
3	0.901	0.802	0.952	0.896	0.794	0.949
4	0.953	0.904	0.957	0.910	0.820	0.956
5	0.864	0.734	0.933	0.971	0.939	0.986
6	0.954	0.905	0.978	0.921	0.840	0.961
7	0.940	0.878	0.971	0.822	0.659	0.911
8	0.905	0.810	0.953	0.893	0.787	0.947
9	0.860	0.727	0.931	0.902	0.804	0.952
10	0.874	0.753	0.938	0.937	0.871	0.969
11	0.920	0.,839	0.961	0.921	0.841	0.962
12	0.869	0.743	0.935	1	1	1
13	0.925	0.848	0.963	0.843	0.697	0.922
14	0.935	0.868	0.968			
15	0.933	0.865	0.968			
16	0.885	0.773	0.944			

* ICC = Intraclass Correlation Coefficient;

† CI = Confidence Interval 95%

The cross-sectional study for the evaluation of design validity and reliability was then carried out. 236 nurses from primary and specialised care participated in the study aimed at personnel from the Ferrol Health Area. The response rate of specialised care nurses (80.1%) was slightly higher than that of primary care nurses (76.0%).

In the EFA of the knowledge questionnaire, 4 factors were identified, which explained 80% of the variance: “Training in the management of SVR” (items 3, 4, 5 and 6), “Theoretical framework of SVR management” (items 2, 9 and 12), “Influence of infrequent situations” (items 7 and 8), “Nursing competencies” (items 10 and 11) (Table 3).

**Table 3:** Exploratory factorial analysis of the knowledge questionnaire. Ferrol, Spain, 2017

Item	Factor 1*	Factor 2^†^	Factor 3^‡^	Factor 4^§^	Communality
Is the SVR^||^ a device that is used in patients who need long-lasting venous access?	0.05	0.84^¶^	0.04	0.24	0.87
Is the administration of anti-tumour drugs the most prominent application of SVR^||^?	-0.04	0.95^¶^	0.05	-0.14	0.88
Is it possible to draw blood repeatedly using the SVR^||^?	0.87^¶^	-0.13	0.12	0.15	0.83
Is it necessary to wash the SVR^||^ after administering blood products?	0.87^¶^	-0.01	0.11	0.12	0.89
Is it necessary to perform heparinization on a regular basis even if the SVR^||^ is not being used?	0.84^¶^	0.11	-0.15	-0.04	0.75
Is it always necessary to insert the needle into the SVR^||^ using a sterile technique?	0.65^¶^	0.30	0.06	-0.44	0.69
Is it possible to fix the needle in the SVR^||^ for 12 hours if it is not going to be used in this time?	0.06	0.06	0.94^¶^	0.08	0.95
Is swimming contraindicated in patients with SVR^||^?	-0.03	0.00	0.98^¶^	-0.10	0.95
Are the electromagnetic waves emitted by the microwave detrimental to the proper functioning of the SVR^||^?	-0.04	0.51^¶^	0.26	0.22	0.43
Is body image disorder a nursing diagnosis frequently suffered by SVR^||^ users?	0.09	0.11	-0.07	0.85^¶^	0.82
Do I consider the SVR^||^ of exclusive use of onco-haematological units?	0.43	0.04	0.17	0.58^¶^	0.78
Do I have training on the SVR^||^?	0.45	0.50^¶^	-0.19	0.15	0.73
Do I consider that it is necessary to have training for the management of the SVR^||^?					

* Factor 1 = Training in the management of the subcutaneous venous reservoir ;

† Factor 2 = Theoretical framework of subcutaneous venous reservoir management;

‡ Factor 3 = Influence of infrequent situations;

§ Factor 4 = Nursing skills ;

|| SVR = Subcutaneous Venous Reservoir;

¶ Burdens that characterize each factor

To make the EFA of this questionnaire, the question 13 was not considered since it is not exactly a knowledge question, but a question about the opinion regarding the importance of personal knowledge, although this does not mean that it is not interesting for the study. In the EFA of the attitude questionnaire, 4 factors that explained 64% of the variance were extracted: “Insecurity in the handling of SVR” (items 1, 6, 7, 9, 10 and 11); “Loss of autonomy in decision-making” (items 2 and 5); “ Arousal of conflicts in the working environment “ (items 14, 15 and 16) and “Bonding to the workplace” (items 3 and 4) (Table 4). Items 8, 12 and 13, which exhibited communalities less than 0.4, were eliminated in the final version of the questionnaire.

**Table 4:** Exploratory factorial analysis of the attitude questionnaire. Ferrol, Spain, 2017

Item	Factor 1*	Factor 2^†^	Factor 3^‡^	Factor 4^§^	Communality
I am allowed to use/management of the SVR^||^ in my unit.	-0.69^¶^	0.23	-0.15	0.14	0.56
If I have a patient with SVR^||^, I check with the patient's responsible doctor to see if I can use it.	0.08	0.71^¶^	0.09	-0.03	0.60
In my unit or work center, I have the adequate material resources for the management of the SVR^||^.	-0.09	0.02	0.04	0.75^¶^	0.58
The protocol for the use and handling of the SVR^||^ is available to me in my unit or work center.	0.07	-0.01	-0.02	0.76^¶^	0.59
I use a peripheral pathway if the patient's attending doctor considers that I should not use the SVR^||^ for a purpose different from the one that caused the indication.	0.00	0.79|^¶^	0.07	0.02	0.66
In case I need to use the SVR^||^, I advise a nurse who is accustomed to handling this type of device to help me.	0.62^¶^	0.38	-0.07	0.01	0.63
I consider that only personnel accustomed to operate this type of device may operate it.	0.47^¶^	0.46	0.02	0.06	0.59
The fear of damaging the device is one of the reasons why I would not use the SVR^||^.	0.46^¶^	0.45	0.11	-0.03	0.66
I consider it safer for the patient to use a central pathway in front of the SVR^||^.	0.77^¶^	-0.02	0.14	0.14	0.75
I'd rather use a peripheral pathway than handle the SVRS^||^.	0.69^¶^	0.14	0.12	-0.08	0.68
I had a patient with SVR^||^ who didn't allow me to operate the device.	0.06	0.19	0.58^¶^	-0.06	0.48
I have worked in teams where the doctor responsible for the patient has not allowed me to use the SVR^||^.	-0.11	0.30	0.75^¶^	-0.02	0.66
I have worked in teams where my own colleagues have not allowed me to use the SVR^||^.	0.22	-0.20	0.81^¶^	0.08	0.87

* Factor 1 = Unsafe management of the subcutaneous venous reservoir;

† Factor 2 = Loss of decision-making autonomy ;

‡ Factor 3 = Arousal of conflicts in the working environment ;

§ Factor 4 = Bonding to the workplace;

|| SVR = Subcutaneous Venous Reservoir;

¶ Burdens that characterize each factor

Cronbach’s alpha of the knowledge questionnaire was raised to 0.750 of the attitude questionnaire to 0.865.

In its final form, the questionnaire consists of 26 items, 13 of them in the knowledge block, instead of the initial 31. This instrument may be used freely by other authors upon request to the author for correspondence and citing this reference appropriately.

## Discussion

In the review of the literature, we found no evidence of the existence of any instrument to measure the degree of knowledge and attitudes of nurses in the management of SVR. On the other hand, studies contributed to the development of our questionnaire. A study carried out at the *Hospital General Universitario de Valencia* (General University Hospital of Valencia) revealed the reluctance of nurses to use these devices for reasons such as lack of security and training, among others^(^ 27 ^)^. In addition, several studies reviewed about the knowledge of the nursing staff regarding the management of SVR show a deficit of these about the device^(^ 13 ^,^ 15 ^,^ 19 ^)^. Other studies, focused on the well-being of the patients using the device, have contributed with respect to the dissatisfaction or discomfort they feel with the handling of their central pathway by the healthcare professionals who attend them^(^ 28 ^)^. It shows the deficit care of nursing staff^(^ 29 ^)^, and even indicate some possible area of improvement in the practice of the nursing professional^(^ 17 ^,^ 30 ^)^.

For all these reasons, we believe in the need to develop a tool that enables measuring the knowledge and attitudes that nursing professionals express when handling the device.

The validity of the content was determined through an individual evaluation of each item by the group of experts using Lawshe’s formula, obtaining values above the established minimums (CVI of 0.51 for fourteen experts).

Reliability was assessed by calculating the ICC, whose value was greater than 0.75 for all questions (greater than 0.90 for 17 of them). It was also evaluated using Cronbach’s alpha, obtaining 0.818 in the first administration of the questionnaire and 0.819 in the retest. In the study carried out in Ferrol with a larger sample, Cronbach’s alpha rises to 0.865. Something similar happens with the knowledge questionnaire, for which the values of Cronbach’s alpha are 0.608 in the test and 0.642 in the retest of the pilot test, and 0.750 in the final study. The final values of Cronbach’s alpha is considered acceptable.

The main limitation of our study comes from the impossibility of randomising the selection of the sample of the study carried out in the Sanitary Area of Ferrol due to compliance with the Organic Law 15/99 of Data Protection^(^ 26 ^)^. To solve this problem, we had to obtain a sample of the largest possible size, reaching a response rate of 78.4%.

We consider that the results obtained in the validation of our questionnaire show it can be a useful tool to evaluate the knowledge and attitudes regarding the management of SVR in populations analogous to the one that was object of our study.

## Conclusion

It is very important that nursing staff manipulate the subcutaneous venous reservoir (SVR) safely, and, for this purpose, they need specific knowledge and attitudes.

Some studies highlight the problematic use of these devices by the professional nursing group, a situation that also affects the well-being of patients with SVR.

No questionnaires measure the level of knowledge and attitudes of the nursing professional towards the use of SVR. Therefore, a validated tool was developed to measure the level of knowledge and attitudes of the nursing professional towards the use of SVR, both in primary care and in specialized care.
